# Prevalence and Predictors of Burnout Among Elementary and High School Teachers in Canada

**DOI:** 10.1192/j.eurpsy.2026.11322

**Published:** 2026-07-31

**Authors:** B. Agyapong, Y. Wei, V. I. O. Agyapong

**Affiliations:** 1 Psychiatry, University of Alberta, Edmonton; 2 Psychiatry, Dalhousie University, Halifax, Canada

## Abstract

**Introduction:**

Burnout has long been recognized as a critical issue among educators, linked to a range of psychological and physical health problems such as depression. It is commonly associated with chronic occupational stress and is characterized by three dimensions: emotional exhaustion (overwhelming fatigue), depersonalization (cynicism and detachment from work), and reduced professional accomplishment (feelings of ineffectiveness and lack of achievement). These dimensions reflect how individuals experience and respond to stress within their social and professional contexts.

**Objectives:**

This study aimed to assess the prevalence and predictors of the three dimensions of burnout: emotional exhaustion, depersonalization, and reduced professional accomplishment, among elementary and high school teachers in Canada.

**Methods:**

A quantitative cross-sectional design was employed. Data were collected via an online survey between September 1, 2022, and August 30, 2023. The Maslach Burnout Inventory-Educator Survey (MBI-ES) was used to measure burnout, the Brief Resilience Scale (BRS), assessed resilience, and the Perceived Stress Scale (PSS) was utilized to evaluate stress levels. Statistical analyses were conducted using SPSS version 28 (IBM Corp).

**Results:**

Out of 1,912 educators who received the survey link, 780 completed the burnout-related items, yielding a response rate of 41%. The prevalence rates for emotional exhaustion, depersonalization, and reduced professional accomplishment were 76.9%, 23.2%, and 30.8%, respectively. Teachers reporting high stress were significantly more likely to experience: Emotional exhaustion (OR = 6.88; 95% CI: 3.31-14.29), Depersonalization (OR = 2.55; 95% CI: 1.65-3.93), and Reduced professional accomplishment (OR = 2.34; 95% CI: 1.64-3.35).

Low resilience was also a strong predictor of emotional exhaustion (OR = 3.26; 95% CI: 2.00-5.31). Male teachers were 2.4 times more likely to report depersonalization compared to females. Additionally, educators who were partnered/cohabiting or identified as “other” in marital status were 3.5 and 7.3 times more likely, respectively, to experience depersonalization than single participants. Physical Education teachers were 3.8 times more likely to exhibit depersonalization compared to English teachers.

**Conclusions:**

High stress and low resilience significantly contribute to burnout among Canadian teachers. Targeted interventions addressing systemic stressors and fostering resilience are essential to mitigate burnout and enhance educator well-being.

**Disclosure of Interest:**

None Declared

